# ROLES OF COMMUNITY COLLEGES IN TRAINING THE HEALTH CARE WORKFORCE IN THE AGING SOCIETY

**DOI:** 10.1093/geroni/igad104.1690

**Published:** 2023-12-21

**Authors:** Takashi Yamashita

**Affiliations:** University of Maryland, Baltimore County, Baltimore, Maryland, United States

## Abstract

As population aging advances, the need for health, medical and long-term, care necessarily increases. On one hand, certain occupations mostly with routine tasks are likely to be automated by computers and technology over time. On the other hand, the previously estimated job automation risks clearly suggest that health care occupations are less likely to be automated, compared to other industries. For example, while 12-45% of health care occupations could be replaced by technology, over 80% of simple food preparation and entry-level customer service is projected to be automated within a few decades. Thus, the demand to train the health care workforce for the aging population will continue to be high, despite the advancement in computers and technology. Certain health care occupations such as licensed practical nurses, nurse assistants, medical technicians, and home health aides, do not require bachelor’s degrees. Considering the current and projected health care workforce shortage, education and training of the future health care workforce is a critical issue when preparing for growing older populations in the U.S. This study triangulated data on health care occupations, literature on workforce development, and emerging trends on aging-related health and long-term care needs, and critically analyzed the role of community colleges to meet the demands for health care workforce in the U.S. Results showed that the history, geographic locations, and existing health care programs position community colleges as one of the most effective and efficient health care workforce development institutions. Specific examples of health care professional training programs are also evaluated.

